# Comparative Transcriptomic Analysis of Water-Deficit Responses in *Japonica* Hybrid Rice ‘Dianheyou 615’

**DOI:** 10.3390/ijms27167469

**Published:** 2026-08-20

**Authors:** Xiaoli Zhou, Cui Zhang, Junjie Li, Xianyu Wang, Chunli Wang, Fan Luo, Wenfeng Zhang, Changhe Wei, Qian Zhu, Lijuan Chen

**Affiliations:** 1 Rice Research Institute, Yunnan Agricultural University, Kunming 650201, China; 18228767665@163.com (X.Z.);; 2 College of Agricultural Science, Xichang University, Xichang 615013, China; 3 School of Life Sciences, Yunnan University, Kunming 650500, China; 4 The Key Laboratory for Crop Production and Smart Agriculture of Yunnan Province, Yunnan Agricultural University, Kunming 650201, China

**Keywords:** hybrid *japonica* rice (*Oryza sativa* L.), ‘Dianheyou 615’, water-deficit response, RNA-seq analysis, drought adaptation, core drought-response molecular module

## Abstract

Water deficit severely limits rice productivity. The elite Dian (D1)-type hybrid *japonica* rice ‘Dianheyou 615 (ZH1)’ exhibits exceptional drought adaptation in high-altitude rainfed uplands of the Yungui Plateau, yet the underlying molecular mechanisms remain unknown. We compared phenotypic and transcriptomic responses of ZH1 and six other *japonica* cultivars under well-watered and water-deficit conditions. Water-deficit stress significantly impaired agronomic traits across all cultivars; however, ZH1 uniquely maintained relatively stable flag leaf morphology and seed-setting rate, and displayed distinctive stomatal traits, in stark contrast to its parental lines and other cultivars. Transcriptomic profiling at the jointing-to-booting stage defined a core drought response module of 174 conserved genes across all cultivars. Critically, by intersecting 1097 ZH1-specific genes with drought-responsive elements, we pinpointed 15 core, cultivar-specific regulatory genes. These candidates are enriched in functions related to cuticle formation, carbohydrate metabolism, and stress signaling; among them, a DREB transcription factor (*LOC4347618*) is a prime candidate. qRT-PCR validated their expression. Using CRISPR/Cas9-mediated gene editing, we generated homozygous knockout mutants for *LOC4333842*, *LOC4347618*, and *LOC4328441*. Under 20% PEG-6000-simulated drought stress, all three mutant lines showed significantly increased drought susceptibility relative to wild-type controls, confirming the positive regulatory roles of these genes in drought stress tolerance in *japonica* rice. These results establish these three genes as promising targets for molecular breeding aimed at enhancing drought resistance in rice.

## 1. Introduction

Rice (*Oryza sativa* L.) serves as the staple food for more than half of the world’s population, and its stable production is therefore directly linked to global food security [1]. However, with the intensification of global climate change and the expansion of arid and semi-arid regions, water deficit has become one of the most critical environmental constraints limiting rice growth, development, and yield formation [2]. It has been estimated that approximately 50% of global rice cultivation areas are affected by drought to varying degrees, resulting in substantial yield losses [3]. Consequently, elucidating the molecular mechanisms underlying drought tolerance in rice, identifying key drought-responsive genes, and developing water-saving, drought-resistant, and high-yield rice cultivars have become strategic priorities for ensuring sustainable rice production and mitigating future food crises. During long-term evolution, plants have developed sophisticated morphological, physiological, and molecular regulatory networks to adapt to water-deficient environments. These adaptive mechanisms include modulation of root system architecture to enhance water uptake, alteration of leaf morphology and stomatal behavior to reduce transpiration, and activation of physiological and biochemical responses such as osmotic adjustment, reactive oxygen species (ROS) scavenging, and stress signaling pathways [4,5].

At the molecular level, previous studies have identified numerous genes and regulatory pathways involved in drought responses in rice [6,7]. These studies have mainly focused on several key aspects. First, drought signal perception and transduction involve both abscisic acid (ABA)-dependent and ABA-independent pathways, in which transcription factor families such as SNF1-related protein kinases 2 (SnRK2s), dehydration-responsive element-binding protein/C-repeat-binding factors (DREB/CBFs), and NAC (NAM, ATAF, and CUC) proteins function as central regulators [2]. Second, osmoprotectant biosynthesis, including the accumulation of proline, soluble sugars, and late embryogenesis abundant (LEA) proteins, contributes to cellular osmotic balance under drought conditions [8]. Third, cellular structure protection is maintained through aquaporins (AQPs), which regulate transmembrane water transport, and antioxidant enzyme systems, which preserve membrane stability by scavenging excessive ROS [9]. With the rapid development of high-throughput sequencing technologies, particularly RNA sequencing (RNA-seq), researchers have been able to systematically characterize the genome-wide transcriptional landscape of rice under drought stress [4]. Nevertheless, several limitations remain in current studies. Most investigations have focused on a limited number of model cultivars, such as Nipponbare, or on specific local landraces, whereas the drought-tolerance mechanisms of widely cultivated elite hybrid rice cultivars remain insufficiently explored [10]. Moreover, many studies have been designed as comparisons within a single genotype under different treatments, lacking systematic multi-genotype analyses. This limitation makes it difficult to distinguish universally conserved “core” drought-response mechanisms from cultivar-specific regulatory modules underlying superior drought-tolerance traits [11].

Substantial genetic variation in drought tolerance exists among different rice cultivars. Hybrid rice varieties, particularly ‘Dianheyou 615 (ZH1)’, a Dian-type (cytoplasmic male sterility type) three-line hybrid cytoplasmic male sterility type *japonica* rice cultivar, generally exhibit superior yield potential and enhanced environmental adaptability owing to strong heterosis [12]. Such advantages may arise from the aggregation of favorable parental alleles and/or novel interaction effects generated through hybridization. Therefore, elucidating the drought tolerance mechanisms of elite hybrid rice cultivars has direct implications for future breeding programs aimed at improving both productivity and stress resistance. In this context, we propose a central scientific question: among the numerous drought-responsive genes, which constitute the conserved core responsive genes shared across *japonica* rice cultivars that form the fundamental framework of drought adaptation? More importantly, which genes function as cultivar-specific regulators unique to elite cultivars such as ZH1, thereby conferring superior drought tolerance compared with other cultivars? Distinguishing these two categories of genes is of great importance for breeding applications, as the former may serve as universal targets for broad-spectrum drought-resistance breeding, whereas the latter may enable precise genetic improvement of elite agronomic traits [12].

Due to its unique adaptability to both irrigated and rainfed conditions, ZH1 is a widely cultivated hybrid *japonica* rice cultivar in the Yungui Plateau of southwestern China, and is recognized for its high yield, yield stability, superior grain quality, and broad-spectrum stress resistance. In agricultural production, ZH1 has demonstrated efficient water utilization and strong tolerance to seasonal drought conditions [13]. However, the molecular regulatory network underlying its superior drought tolerance remains poorly understood. To address this issue, the present study employed ZH1 as the core experimental material and established a multi-genotype comparative framework comprising seven *japonica* rice accessions, including their parental lines, hybrids with contrasting traits, and conventional paddy/upland cultivars. By integrating RNA-seq analysis with detailed agronomic and leaf stomatal trait characterization, this study aimed to achieve three interconnected objectives: (i) to systematically characterize the phenotypic plasticity and transcriptomic response profiles of different *japonica* rice cultivars under water-deficit stress; (ii) to identify and define a set of conserved core drought-responsive genes in *japonica* rice through multi-cultivar comparative analysis, thereby revealing common molecular adaptation strategies; and (iii) to identify ZH1-specific key regulatory factors through fine-scale comparative analyses and dual-filtering strategies, and to elucidate their potential functions. Collectively, this work provides a comprehensive transcriptomic framework for understanding drought adaptation in elite hybrid *japonica* rice and offers a valuable set of candidate genes for future drought-resistance breeding programs [14].

## 2. Results

### 2.1. Water Deficit Induces Differential Phenotypic Responses Among Japonica Rice Cultivars

**Figure 1:**
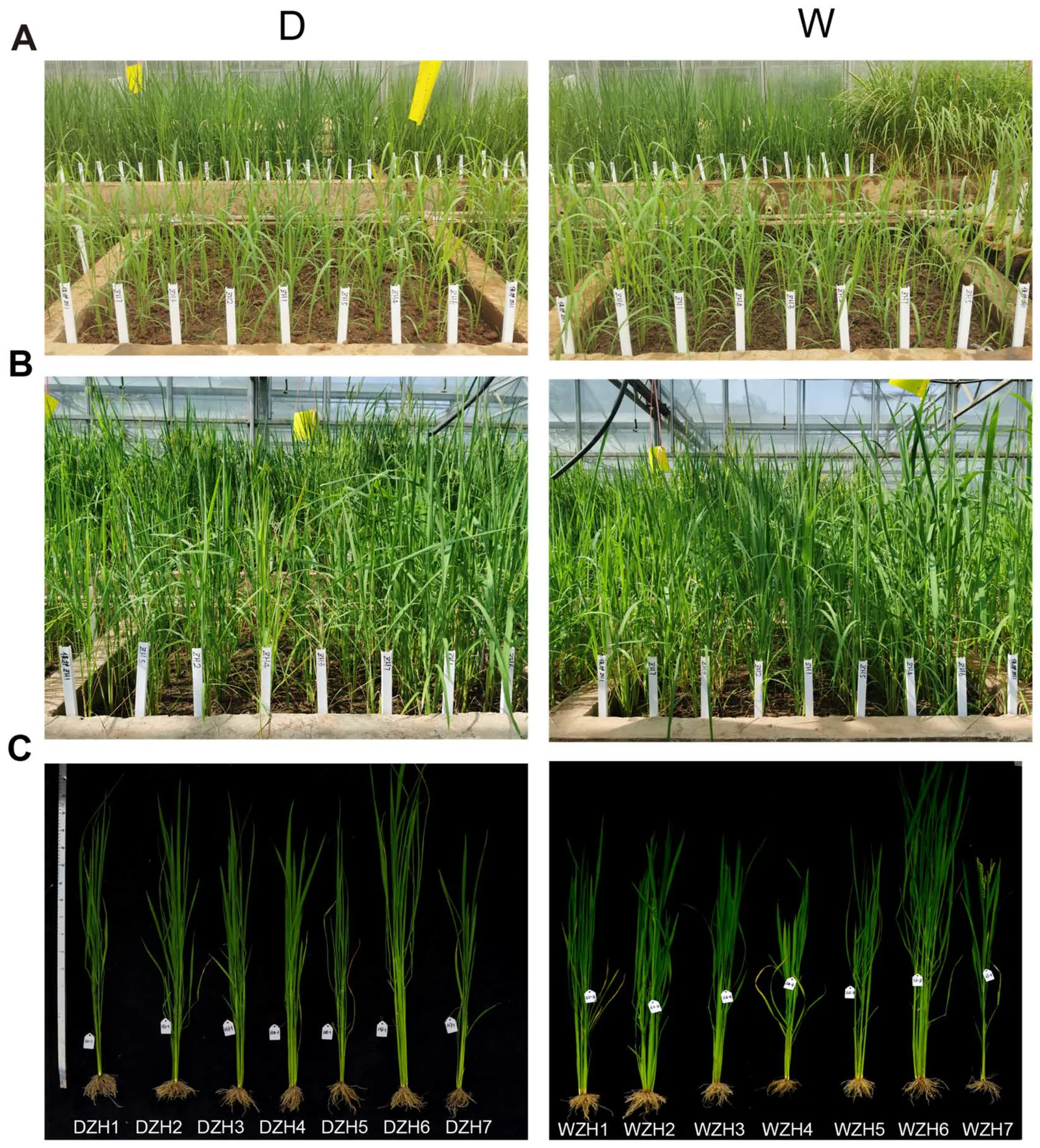
Plant morphological performance of seven rice cultivars under well-watered (W) and water-deficit (D) conditions. (**A**) Rice plants at 45 days after sowing; (**B**) rice plants at 80 days after sowing; (**C**) rice plants at 90 days after sowing.

To evaluate the differential responses of *japonica* rice cultivars to water deficit, twelve key agronomic and physiological traits (Table 1, Table 2) and stomatal morphological characteristics (Table 3) of seven *japonica* accessions were measured under well-watered (W) and drought stress (D) treatments. Overall, water deficit significantly suppressed growth- and yield-related traits as well as stomatal microstructure in most rice cultivars (Figure 1). Compared with the well-watered condition, the number of effective panicles per plant, grain number per panicle, and 1000-grain weight decreased to varying degrees across all cultivars, with the maximum reduction rate of effective panicles reaching 53.3%, and the differences among cultivars being statistically significant (*p* < 0.05 for most traits, as shown in Table 2), indicating substantial genotypic variation in drought adaptability.

Integrating these agronomic and stomatal traits, the hybrid ZH1 exhibited strong heterosis over its parents ZH4 and ZH5. For example, under well-watered conditions, its grain number per panicle (165.22 ± 43.71) was significantly higher than that of ZH4 (102.56 ± 25.47) and ZH5 (150.56 ± 39.77) (*p* < 0.05). Under drought, its seed setting rate (94.96 ± 4.60%) did not differ significantly from the well-watered treatment (96.61 ± 2.30%, *p* > 0.05), whereas those of ZH4 and ZH5 dropped significantly (*p* < 0.05). Under well-watered conditions, ZH1 significantly outperformed both parents in grain number per panicle (165.22 ± 43.71), 1000-grain weight (24.73 ± 0.78 g), and seed setting rate (96.61 ± 2.30%). Its higher chlorophyll SPAD value at the jointing-to-booting stage (40.73 ± 2.71) ensured continuous photosynthate supply for grain filling. Under water deficit, although its grain number decreased to 131.78 ± 39.06 and 1000-grain weight to 22.33 ± 1.89 g, the seed setting rate remained remarkably stable (94.96 ± 4.60%), decreasing only marginally. In stark contrast, the parents ZH4 and ZH5 suffered severe yield losses with sharply reduced seed setting rates under drought. Moreover, the flag leaf chlorophyll SPAD of ZH1 significantly increased under drought (from 40.73 ± 2.71 to 42.59 ± 2.47), suggesting that photosynthetic pigments in its leaves were less prone to degradation, thus sustaining photosynthetic capacity to supply assimilates for grain filling.

Panicle and flag leaf morphological traits jointly supported the drought tolerance and stable yield of ZH1. After drought treatment, the flag leaf width of ZH1 increased from 0.97 ± 0.16 cm to 1.15 ± 0.05 cm, which helped retain sufficient photosynthetic area. The panicle neck length was only slightly shortened, ensuring unobstructed translocation of photosynthates to panicles and alleviating drought-induced blockage of assimilate transport.

Stomatal microstructure further elucidated the drought-tolerant mechanism of ZH1 (Figure S1). Under drought stress, the stomatal aperture of ZH1 showed no significant change (*p* > 0.05), increasing from 0.40 ± 0.11 to 0.44 ± 0.11, which contrasted with most cultivars that exhibited significant reductions in aperture (*p* < 0.05). More importantly, compared with its parents—which displayed narrowed stomata and unstable epidermal structures under drought—ZH1 maintained slightly enlarged stomatal apertures and stable surrounding papillae, facilitating a balance between CO_2_ uptake and moderate water conservation. The coordination of photosynthesis and water retention via epidermal morphology served as a critical morphological basis for stable yield under water deficit.

### 2.2. Transcriptomic Responses to Water-Deficit Stress

**Figure 2:**
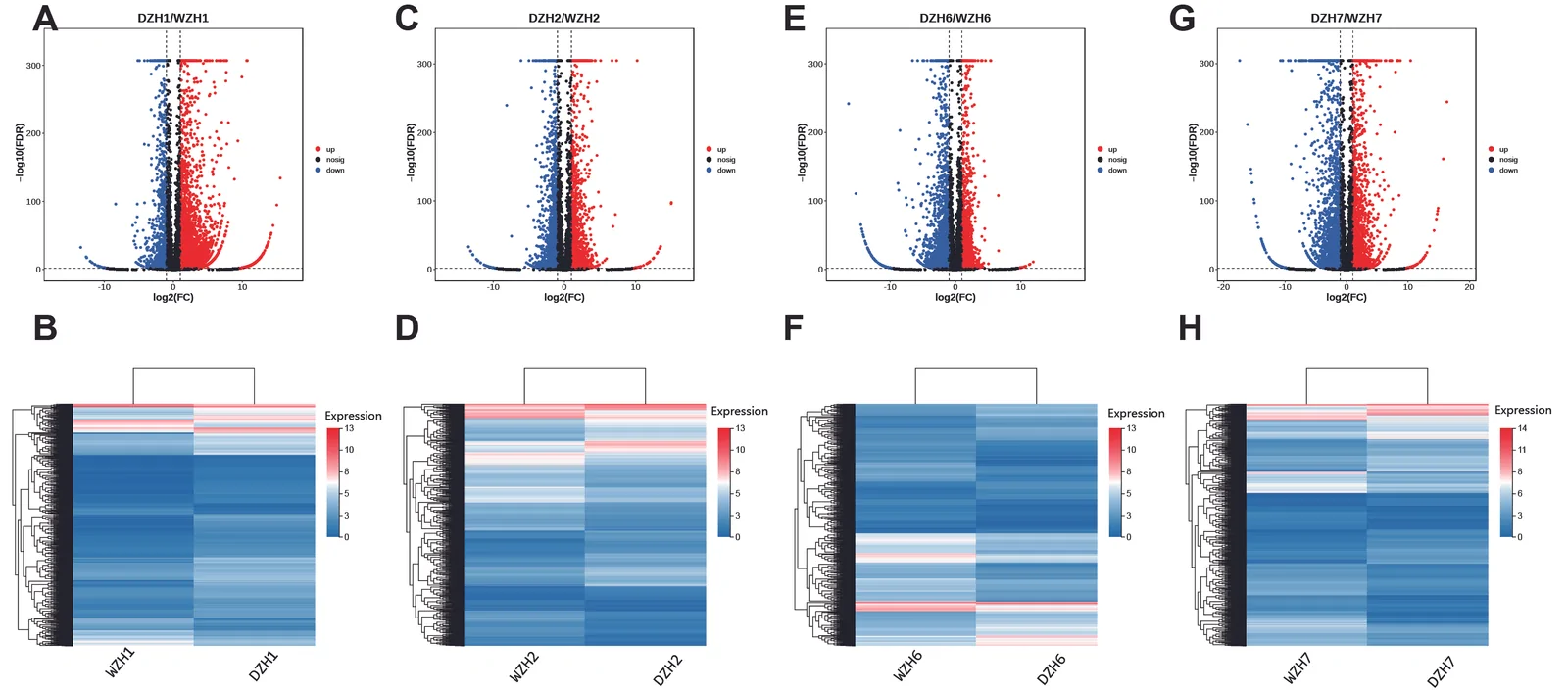
Transcriptomic responses to water-deficit stress in *japonica* rice cultivars. (**A**,**B**) Volcano plot and hierarchical clustering heatmap of DEGs in ZH1 under water deficit (D) versus well-watered (W) conditions. Red and blue dots represent significantly up-regulated and down-regulated genes, respectively (|log_2_FC| ≥ 1, FDR ≤ 0.05). A total of 10,593 DEGs (7784 up, 2809 down) were identified. (**C**,**D**) Volcano plot and heatmap for ZH2, showing 8719 DEGs (3425 up, 5294 down). (**E**,**F**) Volcano plot and heatmap for ZH6, showing 8761 DEGs. (**G**,**H**) Volcano plot and heatmap for ZH7, showing 10,083 DEGs.

To characterize transcriptomic changes in plants under water-deficit conditions, RNA sequencing was performed on seven *japonica* rice cultivars under well-watered (W) and water-deficit (D) conditions at the jointing to booting stage. Differential expression analysis was conducted by comparing D and W samples within each cultivar. In ZH1, a total of 10,593 DEGs were identified, including 7784 upregulated and 2809 downregulated genes (Figure 2A,B). In ZH2, 8719 DEGs were detected, comprising 3425 upregulated and 5294 downregulated genes (Figure 2C,D). Similarly, 8761 DEGs were identified in ZH6 (Figure 2E,F), and 10,083 DEGs were detected in ZH7 (Figure 2G,H). Volcano plots illustrate the distribution of DEGs in each cultivar, and hierarchical clustering analysis showed clear separation between water-deficit and well-watered samples within each cultivar. These results indicate that water deficit induces substantial transcriptional reprogramming across all cultivars, although the number and direction of DEGs differ among genotypes.

### 2.3. Conserved Core Water-Deficit-Responsive Genes Across Multiple Japonica Cultivars

**Figure 3:**
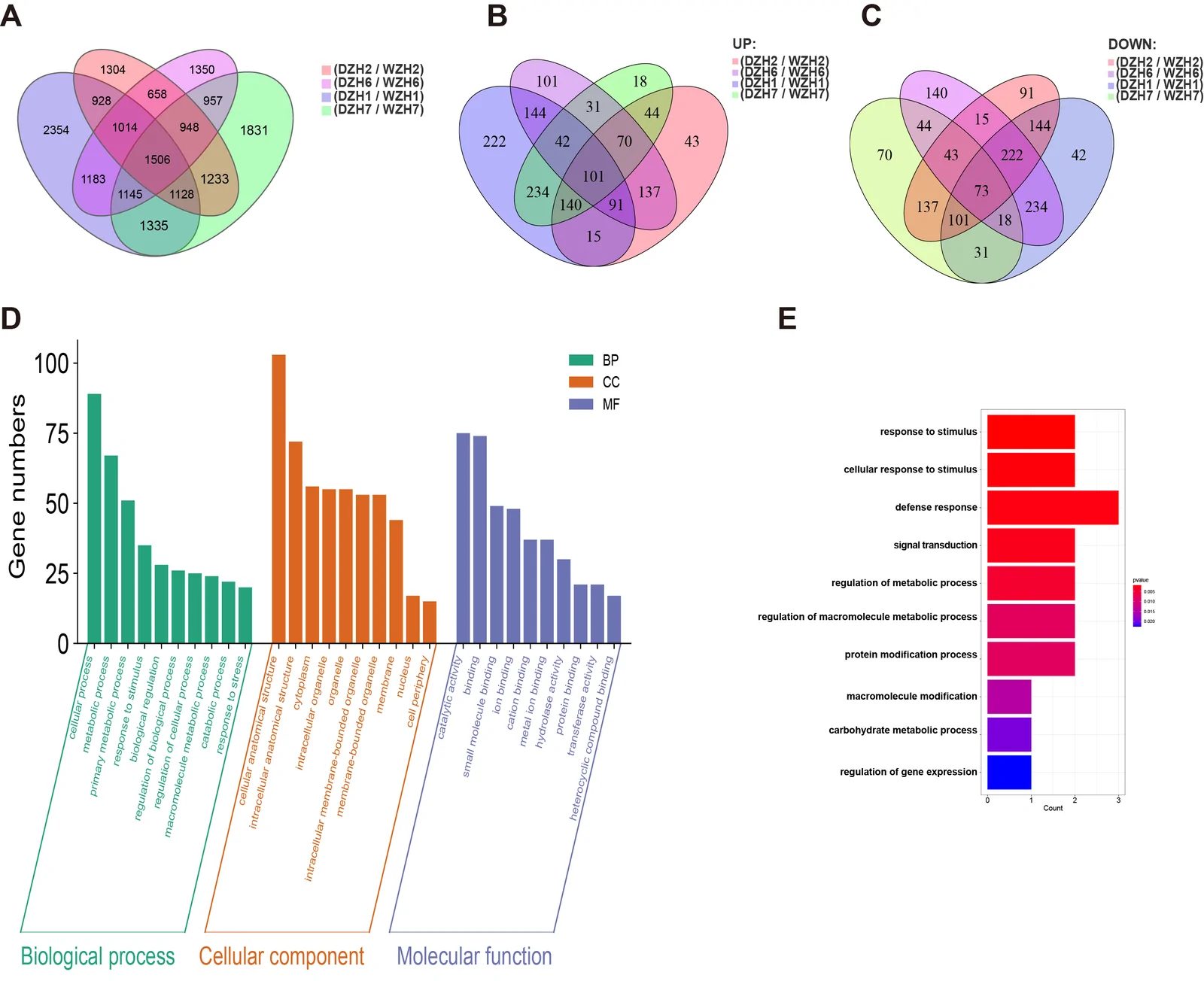
Transcriptomic response and identification of conserved core water-deficit-responsive genes. (**A**) Venn diagram showing the numbers of DEGs under water deficit across four representative cultivars, including ZH1, ZH2, ZH6, and ZH7. (**B**,**C**) Numbers of up-regulated and down-regulated genes in the intersections. (**D**) Bar plots showing the top enriched GO terms in BP, CC, and MF categories for the 174 core water-deficit-responsive genes. (**E**) KEGG enrichment analysis bar chart.

To identify genes commonly involved in water-deficit responses, DEGs derived from four representative cultivars, ZH1, ZH2, ZH6, and ZH7, were intersected. This comparison revealed 1506 genes that were differentially expressed under water-deficit conditions in all four cultivars. A hypergeometric test confirmed that this overlap was significantly enriched (*p* < 0.001), indicating a non-random core responsive module. In addition, 2354 genes were uniquely responsive to water deficit in ZH1 (Figure 3A). Among these shared DEGs, 174 genes exhibited consistent directional expression changes. Among them, there are 101 up-regulated genes and 73 down-regulated genes (Figure 3B,C). These genes were defined as core water-deficit-responsive genes, representing a conserved transcriptional module associated with drought response in *japonica* rice. This conserved gene set provides a foundation for dissecting both shared and cultivar-specific drought-adaptation mechanisms.

To elucidate the biological functions of the 174 core water-deficit-responsive genes, GO and KEGG pathway enrichment analyses were performed using the Database for Annotation, Visualization and Integrated Discovery (DAVID). These genes were significantly enriched in 603 biological processes (BP), 127 cellular components (CC), and 623 molecular functions (MF) categories, as well as 53 KEGG pathways. The top enriched GO terms were primarily associated with metabolic processes, stress responses, energy metabolism, and redox-related processes, while CC terms mainly involved cytoplasmic and membrane-associated cellular components. MF analysis revealed significant enrichment in catalytic activity, binding, small molecule binding, and ion-binding functions, indicating extensive enzymatic and regulatory activity under water-deficit conditions (Figure 3D). KEGG pathway analysis further identified drought-relevant pathways, including plant hormone signal transduction, secondary metabolite biosynthesis, stress response, and carbohydrate metabolism (Figure 3E). Collectively, these enrichment results indicate that the core genes coordinately regulate metabolic reprogramming, energy allocation, and stress signaling to maintain cellular homeostasis and enhance *japonica* rice adaptation to water-deficit stress.

### 2.4. Identification of ZH1-Specific Transcriptional Features Under Different Water Regimes

**Figure 4:**
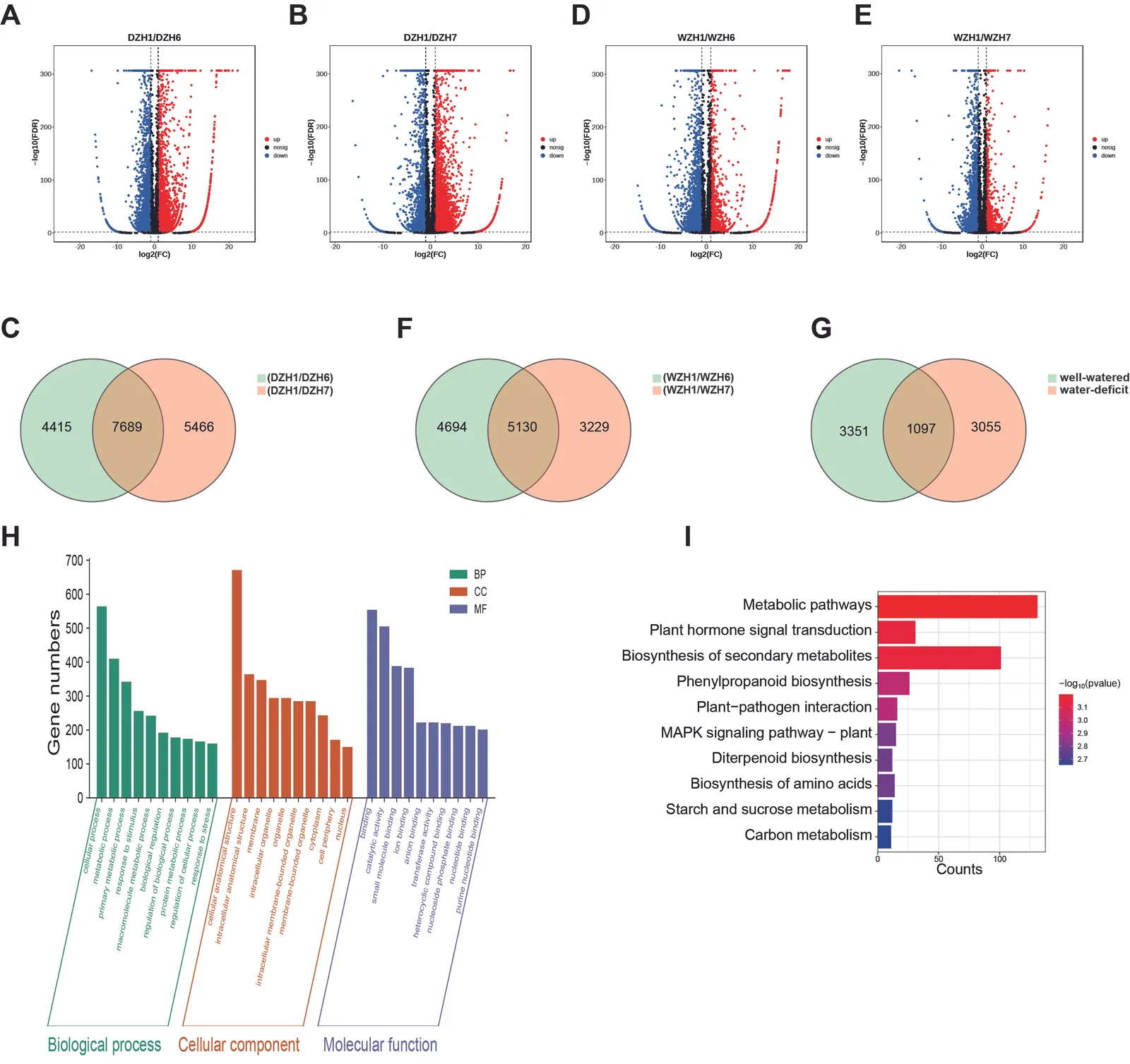
Cultivar-specific transcriptional features of ZH1. (**A**) Volcano plot of DEGs between ZH1 and ZH6 under water-deficit conditions (DZH1 vs. DZH6). (**B**) Volcano plot between ZH1 and ZH7 under water-deficit conditions (DZH1 vs. DZH7). (**C**) Venn diagram showing the intersection of DEGs from (**A**) and (**B**), yielding 7689 ZH1-associated genes. (**D**,**E**) Volcano plots for comparisons under well-watered conditions (WZH1 vs. WZH6 and WZH1 vs. WZH7). (**F**) Intersection of well-watered comparisons, yielding 5130 shared genes. (**G**) Intersection of water-deficit and well-watered cultivar-specific gene sets, identifying 1097 ZH1-specific genes. (**H**,**I**) GO and KEGG enrichment analyses of the 1097 ZH1-specific genes, highlighting pathways involved in metabolism, hormone signaling, secondary metabolism, and stress responses.

To characterize transcriptional features specific to ZH1, pairwise differential expression analyses were conducted between ZH1 and two reference cultivars, ZH6 and ZH7, under both water-deficit and well-watered conditions. Under water-deficit conditions, comparisons between ZH1 and ZH6 and between ZH1 and ZH7 identified 12,104 and 13,155 DEGs, respectively (Figure 4A,B). Intersection of these two DEG sets yielded 7689 genes that were consistently differentially expressed in ZH1 relative to both reference cultivars (Figure 4C). Among these, 4152 genes displayed consistent expression trends, indicating stable cultivar-specific transcriptional regulation under water-deficit conditions. Under well-watered conditions, comparisons between ZH1 and ZH6 and between ZH1 and ZH7 each identified 9824 and 9824 DEGs (Figure 4D,E). The intersection of these DEGs resulted in 5130 shared genes, among which 4448 genes showed consistent expression trends across both comparisons (Figure 4F). To identify transcriptional features that consistently distinguish ZH1 regardless of water availability, the cultivar-specific gene sets obtained under water-deficit and well-watered conditions were intersected. This analysis yielded 1097 genes that were consistently differentially expressed in ZH1 across both water regimes (Figure 4G). To investigate the functional characteristics of genes specifically associated with ZH1, GO and KEGG enrichment analyses were performed for the 1097 cultivar-specific genes using the DAVID database. These genes were significantly enriched in 1423 BP, 260 CC, and 726 MF categories, as well as 112 KEGG pathways. The top enriched terms and pathways were mainly related to metabolic regulation, energy supply, plant hormone signaling, secondary metabolite biosynthesis, and stress-responsive signaling pathways (Figure 4H,I). In addition, several enriched pathways were associated with carbon and nitrogen metabolism and starch biosynthesis. indicating a tight coordination between stress adaptation and fundamental physiological processes.

### 2.5. Integrated Analysis Identifies 15 Core Regulators of Water-Deficit Adaptation in ZH1, Including 13 Cultivar-Specific and Two Japonica-Conserved Genes

**Figure 5:**
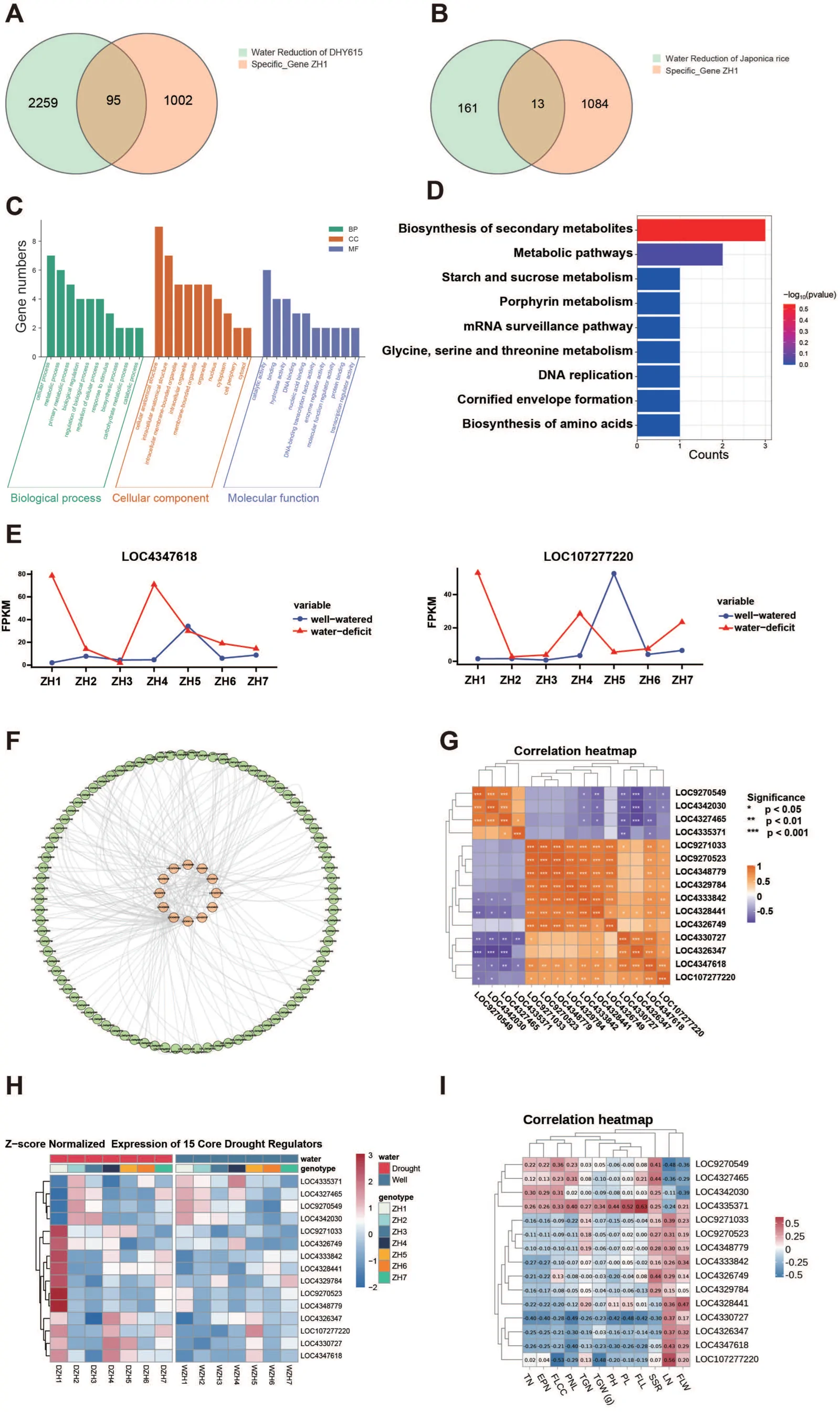
Integrated analysis identifies 15 core regulators of water-deficit adaptation in ZH1. (**A**) Venn diagram intersecting ZH1-specific water-responsive genes (2354) with total ZH1-specific genes (1097), resulting in 95 candidate genes. (**B**) Intersection of conserved core water-deficit-responsive genes (174) with ZH1-specific genes (1097), yielding 13 ZH1-specific core candidates. Two additional *japonica*-conserved genes (*LOC107277220* and *LOC4347618*) were included, forming a final set of 15 core regulators. (**C**) GO enrichment of the 15 genes across BP, CC, and MF categories. (**D**) KEGG pathway enrichment showing the top pathways (e.g., starch/sucrose metabolism, cuticle formation, glycine metabolism). (**E**) Expression profiles of *LOC107277220* and *LOC4347618* across all cultivars and treatments (W: well-watered; D: water deficit). (**F**) Heatmap of predicted upstream transcription factors (MYB, bHLH, NAC, and WRKY families) binding to the 15 target genes. (**G**) Correlation heatmap of expression patterns for the 15 candidate genes across all samples. Color intensity corresponds to Pearson correlation coefficients; red represents positive correlations and blue represents negative correlations. Asterisks indicate the statistical significance of correlations: * *p* < 0.05, ** *p* < 0.01, *** *p* < 0.001. (**H**) Heatmap showing gene-wise Z-score normalized VST expression profiles of 15 core drought-responsive genes across all samples. Columns are grouped by water regime, with a vertical gap separating well-watered and drought-treated samples. Genotype and water treatment information is annotated at the top. (**I**) Correlation heatmap illustrating pairwise associations between expression levels of 15 core drought-responsive genes and physiological agronomic traits. Pearson correlation coefficients are indicated by color gradient.

To comprehensively identify genes that are both responsive to water deficit and uniquely associated with ZH1′s adaptive capacity, we performed an integrative analysis combining multiple gene sets. First, by intersecting the 2354 ZH1-specific drought-responsive genes with the 1097 ZH1-specific constitutive genes (Figure 5A). Second, taking the common water-related genes shared by *japonica* rice and the unique genes of ZH1, the intersection was obtained, resulting in 13 genes in ZH1 that have different water-reduction mechanisms compared to the other strains (Figure 5B). Functional enrichment analysis of these 15 genes revealed significant associations with chlorophyll catabolism, carbohydrate metabolic processes, cuticle formation, and transcriptional regulation (Figure 5C,D). From the 174 conserved core genes, we further identified two genes (*LOC107277220* and *LOC4347618*) that exhibited particularly strong and consistent differential expression in ZH1, and thus were included as conserved regulators with cultivar-enhanced responsiveness. (Figure 5E). *LOC4347618* encodes a DREB-family transcription factor, a known master regulator of abiotic stress responses.

We then selected 13 genes with known or predicted functions in drought response, including pathways such as glycine metabolism, cell wall biosynthesis, and secondary metabolism. These 13 genes were designated as ZH1-specific core candidates (*LOC4335371*, *LOC9270549*, *LOC4342030*, *LOC4326347*, *LOC4327465*, *LOC4330727*, *LOC4328441*, *LOC4333842*, *LOC4348779*, *LOC9270523*, *LOC4329784*, *LOC9271033*, *LOC4326749*). By combining the 13 ZH1-specific genes with the two *japonica*-conserved genes, we obtained a final set of 15 core regulators underlying water-deficit adaptation in ZH1.

To further explore the upstream regulatory landscape, we predicted potential transcription factors (TFs) binding to the promoter regions of these 15 genes using the PlantRegMap database. A total of 84 putative TFs were identified for 15 target genes, belonging to families such as MYB, bHLH, NAC, and WRKY (Figure 5F). Correlation analysis of the expression profiles of the 15 genes across all cultivars and treatments revealed a strong correlation (Figure 5G), indicating functional coherence among these regulators. Collectively, these results define a multi-layered transcriptional regulatory network that integrates conserved and cultivar-specific components to confer drought adaptation in ZH1.

Pearson correlation analysis between the expression levels of the 15 core candidate genes and agronomic traits (Figure 5H,I) revealed that most drought-responsive genes were strongly correlated with flag leaf width and leaf number (|r| > 0.35 for multiple genes), whereas correlations with grain number per panicle and 1000-grain weight were comparatively weak. Partial correlations were also observed with seed-setting rate and flag leaf chlorophyll content. Given that flag leaf width determines photosynthetic area and leaf number reflects vegetative growth capacity, these findings suggest that the identified core regulators primarily enhance drought adaptation by modulating leaf morphology and sustaining source-organ integrity, thereby indirectly stabilizing grain sink capacity under stress.

### 2.6. Transcriptional Divergence Between ZH1 and Its Parental Lines Reveals the Molecular Basis of Heterosis and Drought Resistance

**Figure 6:**
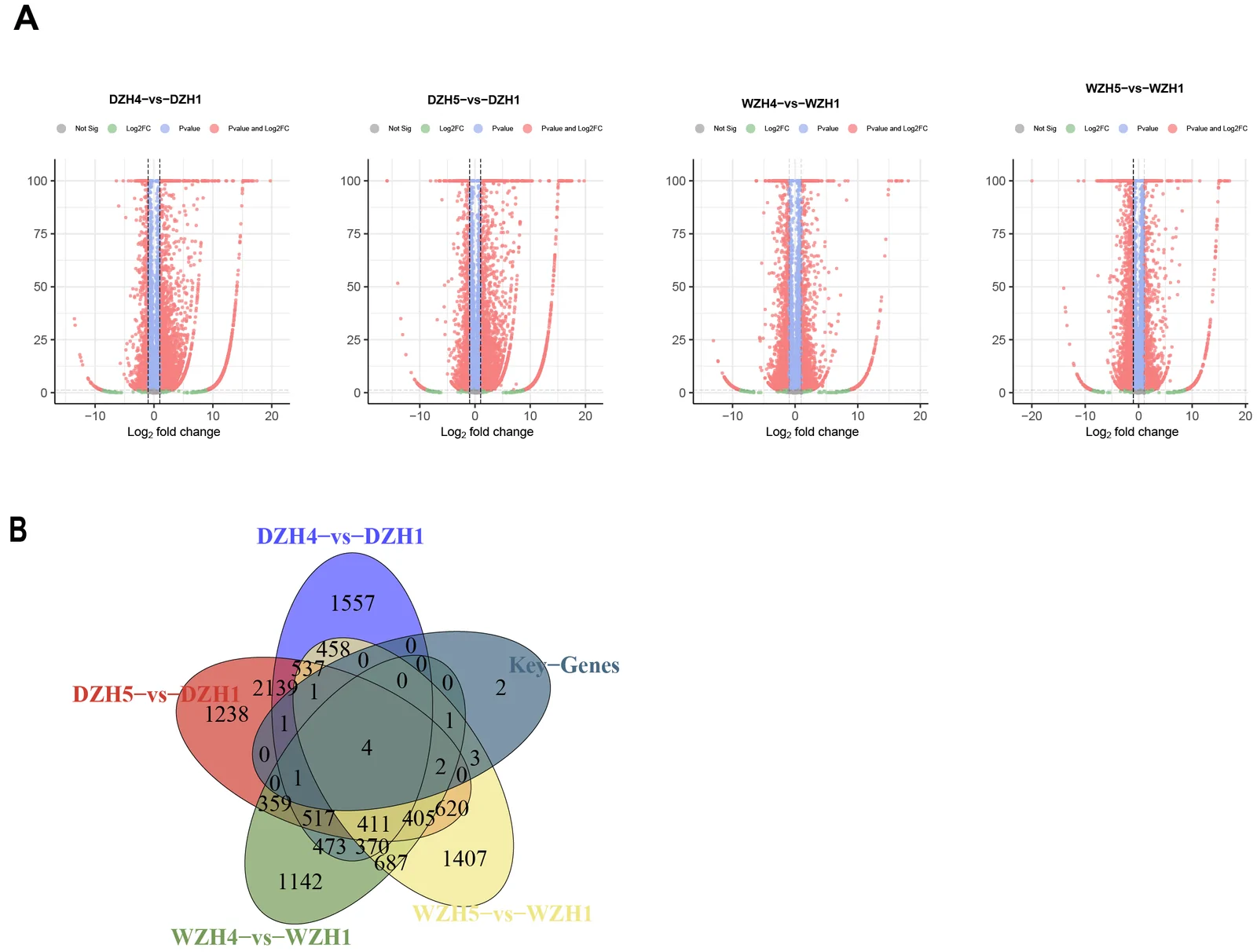
Transcriptomic comparison between ZH1 and its two parental lines under well-watered and water-deficit conditions. (**A**) Volcano plots showing DEGs derived from four pairwise transcriptomic comparisons: ZH1 vs. ZH4 under well-watered treatment, ZH1 vs. ZH5 under well-watered treatment, ZH1 vs. ZH4 under water-deficit treatment, and ZH1 vs. ZH5 under water-deficit treatment. Red dots represent significantly up-regulated genes, and blue dots represent significantly down-regulated genes (|log_2_(fold change)| ≥ 1.0, FDR ≤ 0.05). (**B**) Venn diagram illustrating the overlap of the 15 core drought regulatory genes across the four comparison groups, identifying four genes with consistent differential expression and two genes without significant expression variation between ZH1 and its parental lines.

To clarify the transcriptional heterosis supporting superior drought tolerance of ZH1, pairwise transcriptome comparisons between ZH1 and its two parents (ZH4, ZH5) were conducted separately under well-watered and water-deficit treatments (Figure 6A). Each comparison group yielded more than 1000 DEGs, indicating extensive transcriptional divergence between the hybrid and its parents under both water regimes. Among the 15 core drought regulatory genes identified in this study, four genes (*LOC4333842*, *LOC9270523*, *LOC4326749*, *LOC4348779*) exhibited significant expression differences across all four cross-comparisons, whereas only two genes (*LOC4327465*, *LOC4335371*) showed no obvious expression variation between ZH1 and its parents (Figure 6B). Such widespread transcriptional divergence and consistent differential expression of most core genes fully demonstrate prominent transcriptional heterosis derived from parental genetic recombination. The four stably divergent core genes serve as central modulators that continuously optimize yield components, leaf photosynthetic capacity, and stomatal epidermal structure under both sufficient and limited water supply. The transcriptional reprogramming mediated by these genes coordinately regulates carbohydrate metabolism, cuticle formation, and stress signal cascades, collectively explaining why ZH1 possesses stronger water retention capacity, milder yield decline, and overall superior drought adaptability relative to its two parental lines.

### 2.7. qRT-PCR Validation of 15 Gene Expressions

**Figure 7:**
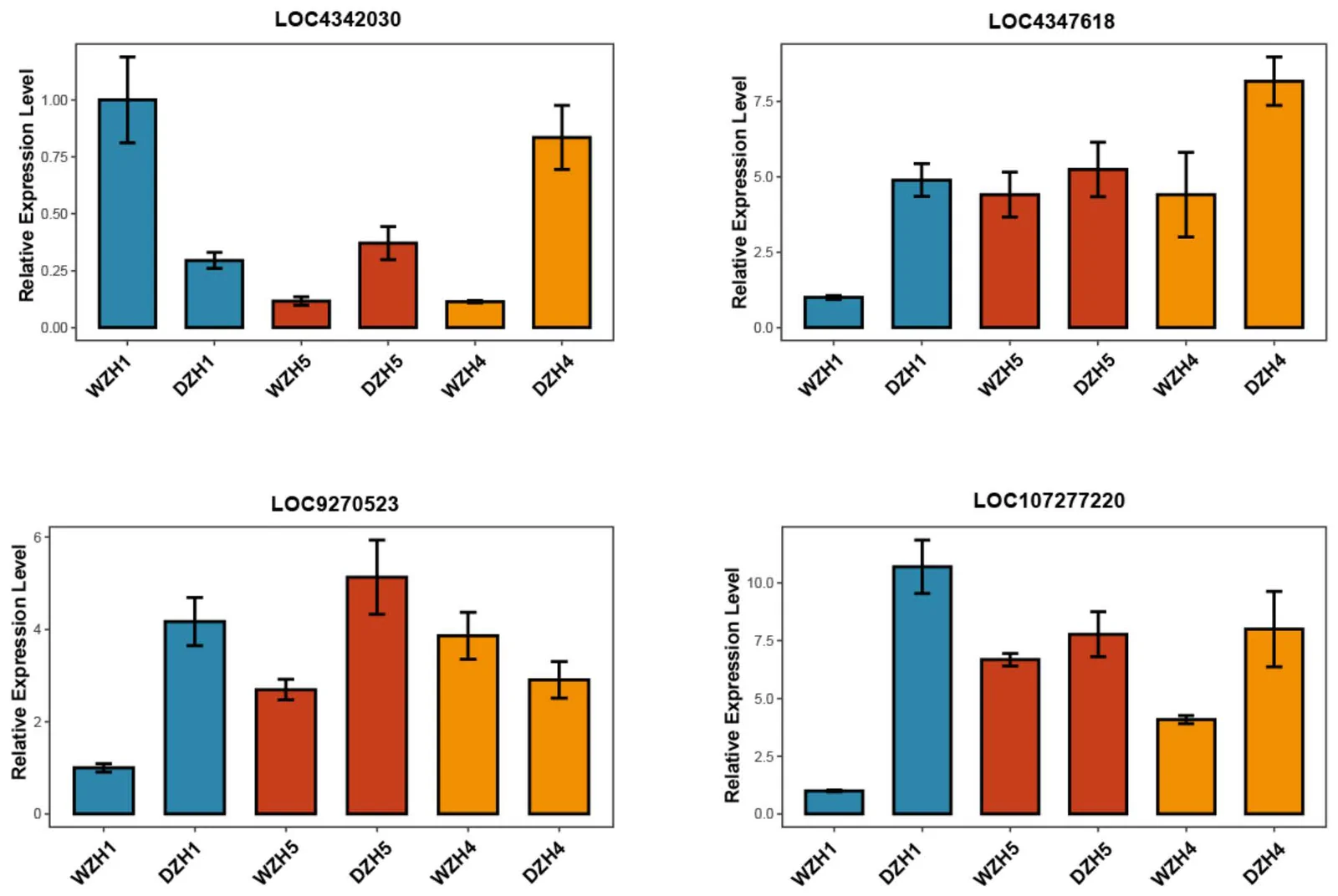
qRT-PCR validation of expression patterns of candidate genes associated with drought response.

To further validate the reliability of the RNA-seq data, qRT-PCR was performed on the 15 identified candidate genes in ZH1 and its parental lines under well-watered (W) and water-deficit (D) conditions (Figure 7, Figure S2).

Overall, the expression patterns obtained from qRT-PCR were largely consistent with the RNA-seq results, supporting the robustness of the transcriptomic analysis. Among the selected genes, several exhibited significant upregulation under water-deficit conditions, including *LOC4347618*, which showed a pronounced increase in expression in ZH1 compared with control conditions. In contrast, genes such as *LOC4342030* displayed downregulation under water deficit, consistent with transcriptome-derived trends.

In addition, most candidate genes showed similar directional changes across biological replicates, although the magnitude of expression varied among genotypes. This variation is consistent with differences in genetic background and stress responsiveness among cultivars. These results confirm the reliability of the RNA-seq data and support the validity of the identified candidate genes associated with drought response in ZH1.

Relative expression levels of 4 selected candidate genes were determined by qRT-PCR in ZH1 and its parental lines under well-watered (W) and water-deficit (D) conditions.

### 2.8. Functional Validation of Core Candidate Genes via CRISPR/Cas9-Mediated Mutagenesis

**Figure 8:**
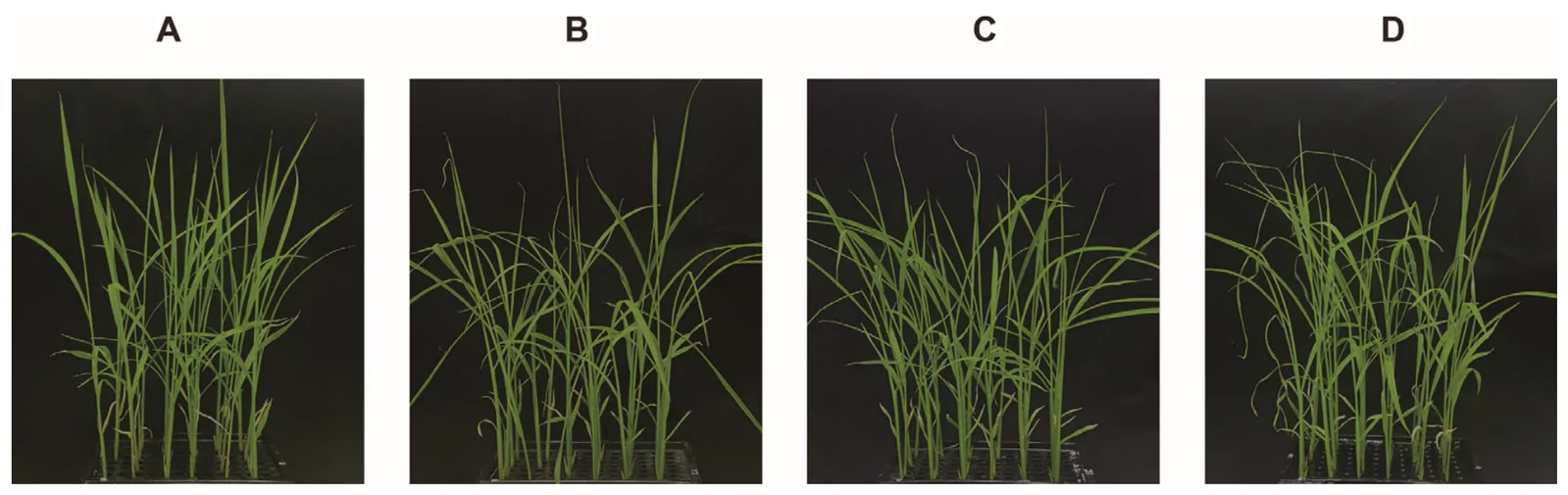
Phenotypic comparison of wild-type (ZH5) and three homozygous knockout lines under 20% PEG-6000-simulated drought stress. (**A**) Wild-type ZH5. (**B**) *LOC4333842* knockout line (T_1_ generation). (**C**) *LOC4347618* knockout line (T_1_ generation). (**D**) *LOC4328441* knockout line (T_1_ generation).

To functionally validate the drought-responsive roles of the identified candidate genes, we generated homozygous knockout mutants for three core candidate genes (*LOC4333842*, *LOC4347618*, and *LOC4328441*) using the Clustered Regularly Interspaced Short Palindromic Repeats/CRISPR-associated protein 9 (CRISPR/Cas9) system. Under 20% (*w*/*v*) PEG-6000-simulated drought stress, all three mutant lines displayed significantly enhanced drought sensitivity relative to wild-type controls, as evidenced by more severe leaf rolling and wilting (Figure 8). These findings confirm that *LOC4333842*, *LOC4347618*, and *LOC4328441* function as positive regulators of drought stress tolerance in *japonica* rice.

## 3. Discussion

Understanding the molecular basis of drought stress responses in crops is fundamental to genetic improvement for stress tolerance. Previous studies in *japonica* rice have typically examined single-cultivar responses or employed bulk RNA-seq for DEG screening, without dissecting conserved versus cultivar-specific regulatory modules in a multi-genotype context. This has resulted in large, poorly validated gene sets with limited breeding utility [15,16]. Here, we established an integrated framework encompassing multi-cultivar phenotyping, comparative transcriptomics, and multi-dimensional candidate gene screening. By integrating phenotypic and transcriptomic data from four representative *japonica* cultivars, we not only defined a conserved drought-responsive core module, but also identified 15 ZH1-specific core regulators through stringent cross-screening. The functional relevance of these genes was confirmed by qRT-PCR and CRISPR/Cas9-mediated mutagenesis, effectively bypassing the bottlenecks of candidate gene redundancy and inefficient validation that have constrained previous transcriptomic studies.

### 3.1. Water Deficit Induces Extensive Transcriptomic Reprogramming and Differential Drought-Response Strategies Among Japonica Rice Cultivars

Plant adaptation to drought stress relies on phenotypic plasticity and rapid transcriptomic reprogramming, a regulatory paradigm that has been well documented in multiple species, including Arabidopsis, rice, and maize [17,18]. Previous studies have demonstrated that drought stress significantly suppresses yield-related traits such as effective panicle number, grain number per panicle, and 1000-grain weight in rice, with genotypic differences in drought tolerance being directly correlated with the extent of trait inhibition.

Notably, ZH1 maintained a relatively high grain number per panicle and a stable seed-setting rate under drought stress (with the seed-setting rate decreasing only from 96.61 ± 2.30% to 94.96 ± 4.60%, significantly outperforming the control cultivar ZH6), accompanied by a slight increase in chlorophyll content to sustain photosynthetic supply. The agronomic performance of ZH1 was substantially less suppressed by stress than that of its parental lines and control cultivars. This phenotype is consistent with the previously reported characteristics of drought-tolerant rice cultivars [19]. However, through stomatal micromorphological observation, we found that ZH1 did not adopt the common strategy of “stomatal closure to reduce water loss” typically employed by conventional drought-tolerant varieties. Instead, ZH1 moderately enlarged its stomatal aperture while maintaining a stable number of papillae around stomata, forming an epidermal adaptive trait that balances water retention and photosynthesis. This unique stomatal regulatory pattern not only avoids insufficient photosynthetic carbon assimilation caused by excessive stomatal closure, but also reduces non-stomatal water loss through the specialized epidermal papillae structures, thereby serving as a critical morphological basis for maintaining leaf physiological homeostasis and mitigating yield loss under drought stress. These findings extend the current understanding of stomatal regulation in rice drought tolerance.

Transcriptomically, drought induced extensive reprogramming in all cultivars, with ZH1 showing 10,593 DEGs (7784 up, 2809 down) versus 8719, 8761, and 10,083 in ZH2, ZH6, and ZH7, respectively. While larger DEG numbers have been conventionally interpreted as indicators of greater transcriptional plasticity and tolerance [20], our co-expression network comparisons suggest that tolerance depends not on DEG quantity but on response efficiency and coordination. ZH1 exhibited a “robust and coordinated” pattern—broadly mobilizing stress-responsive genes while maintaining a more finely tuned regulatory network that optimizes the balance between defense and growth, minimizing energy costs from overactivated defense pathways [21,22]. This finding revises the oversimplified view that “more DEGs confer stronger tolerance” and offers new insights into crop transcriptional regulatory strategies under drought.

### 3.2. Conserved Core Drought-Responsive Genes Define the Fundamental Regulatory Framework of Drought Tolerance in Japonica Rice

In the present study, by comparing drought-responsive DEGs across four representative *japonica* cultivars, we identified 174 genes that consistently responded to drought stress in all cultivars (101 upregulated and 73 downregulated), which we defined as the conserved core drought-responsive genes of *japonica* rice. Notably, the size of this gene set was substantially smaller than the total number of DEGs identified in any individual cultivar, suggesting that these 174 genes constitute the most fundamental and streamlined molecular regulatory backbone of drought tolerance in *japonica* rice [23]. Furthermore, core stress-responsive gene sets have also been identified in the *Oryza* genus in previous studies [24]; the genes in our set that did not overlap with previously reported ones may represent *japonica* subspecies-specific drought-responsive genes, warranting further in-depth investigation.

Building upon this conserved gene set, we employed a multi-dimensional cross-screening strategy integrating “ZH1-specific responsive screening, co-expression module association, and phenotype-correlation ranking” to further narrow the candidate gene pool to 15 core genes tightly associated with the specific drought tolerance of ZH1. qRT-PCR validation confirmed that the expression patterns of these 15 genes were highly consistent with the transcriptomic data. Furthermore, CRISPR/Cas9-mediated gene editing and functional validation of three representative genes demonstrated that knockout of each resulted in significantly enhanced drought sensitivity at the seedling stage in *japonica* rice, directly confirming the efficiency and accuracy of our candidate gene screening strategy. In contrast to previous transcriptomic studies that typically yielded hundreds of candidate genes with limited functional validation, the core gene set obtained in this study is both compact in size and rigorously validated, providing well-defined targets for subsequent mechanistic dissection and breeding applications.

### 3.3. Screening Strategy and Biological Interpretation of the ZH1-Specific Regulatory Module

One of the major innovations of this study lies in the implementation of a dual “conserved + specific” screening strategy. To precisely identify the molecular basis underlying the superior drought tolerance of ZH1, we established stringent screening criteria whereby candidate genes were required to simultaneously satisfy two conditions: (i) involvement in drought responses, and (ii) cultivar-specific expression characteristics in ZH1.

The screening workflow was conducted as follows. First, the intersection between ZH1-specific drought-responsive genes (2354 genes) and ZH1-specific constitutively differentially expressed genes (1097 genes showing differential expression relative to control cultivars under both water conditions) yielded 95 candidate genes, from which 13 genes with clear functional annotations were selected. These 13 genes were not only uniquely drought-responsive in ZH1 but also consistently exhibited cultivar-specific expression patterns. Subsequently, integration of these 13 genes with the conserved core gene set (174 genes), together with two conserved *japonica* rice genes (*LOC107277220* and *LOC4347618*), resulted in the identification of 15 core regulatory factors. Among these, 13 genes represented ZH1-specific core candidate genes, whereas two genes were conserved among *japonica* rice cultivars. Collectively, these genes shared two key features: they either participated directly in drought responses or exhibited ZH1-specific responsiveness, and they suggested that they contribute directly to the superior drought-adaptive phenotype of this cultivar.

Functional enrichment analyses further supported this hypothesis. These 15 genes were significantly enriched in pathways associated with chlorophyll catabolism, carbohydrate metabolism, cuticular wax and cuticle formation, and transcriptional regulation. In particular, enrichment of cuticle formation pathways corresponded closely with the ability of ZH1 to maintain flag leaf stability and reduce non-stomatal water loss under drought conditions [25]. Thickening of the cuticle can effectively reduce transpiration rates while maintaining leaf water potential and photosynthetic activity. Furthermore, enrichment of carbohydrate metabolic pathways was closely associated with energy supply and osmotic adjustment. Efficient sugar metabolism not only provides energy for stress defense responses but also contributes to osmotic regulation through soluble sugar accumulation, thereby maintaining cellular turgor pressure. This mechanism is particularly important for sustaining pollen viability and improving fertilization efficiency under stress conditions [25], which may explain the maintenance of high seed-setting rates in ZH1 during drought stress.

### 3.4. Mechanistic Implications of the Candidate Gene LOC4347618 and Its Upstream Regulatory Network

Using the PlantRegMap database, we further predicted transcription factors potentially binding to the promoter regions of these genes in order to elucidate the upstream regulatory mechanisms governing the 15 core regulatory factors. The results indicated that the upstream regulatory network was primarily associated with four major transcription factor families: MYB, bHLH, NAC, and WRKY. These transcription factor families have been widely implicated in plant drought responses. MYB transcription factors regulate cuticle biosynthesis and stomatal development [26,27]; bHLH proteins participate in ABA signaling and osmotic regulation [28]; NAC transcription factors function as central regulators of drought-responsive pathways by activating multiple downstream stress-defense genes [29,30]; and WRKY transcription factors enhance drought tolerance through regulation of reactive oxygen species scavenging and senescence-associated gene expression [31,32]. Correlation analyses demonstrated that the expression patterns of these 15 genes were significantly positively correlated across all cultivars and treatments, suggesting coordinated regulation by a shared upstream transcriptional network and the formation of a functionally synergistic drought-responsive module.

Based on these findings, we propose the existence of a hierarchical transcriptional regulatory cascade in ZH1. In response to drought signals, upstream MYB, bHLH, NAC, and WRKY transcription factors coordinately regulate the expression of the 15 core genes, including *LOC4347618*. Activation of these genes subsequently promotes downstream osmotic adjustment, cuticle biosynthesis, and reactive oxygen species scavenging pathways, thereby generating a cascading amplification effect that ultimately confers the superior drought adaptability observed in ZH1.

### 3.5. Implications for Drought-Resistance Breeding in Japonica Rice and Future Perspectives

The findings of this study provide valuable genetic resources and strategic guidance for molecular breeding of drought-tolerant rice. The set of 174 conserved core genes can be converted into high-throughput molecular markers, such as Kompetitive Allele-Specific PCR (KASP) markers, for rapid screening of drought tolerance potential in rice germplasm resources. Because these genes represent drought-responsive modules shared across all tested *japonica* rice cultivars, they possess broad applicability in breeding programs. In addition, the 15 ZH1 core regulatory factors represent promising targets for molecular design breeding. Precise modification of these genes in elite but drought-sensitive cultivars using gene-editing technologies such as CRISPR-Cas9 may enable targeted enhancement of drought tolerance [33]. Furthermore, integration of these key genes with advantageous agronomic traits of ZH1, such as greater flag leaf width and high seed-setting rate, may facilitate simultaneous improvement of drought resistance and yield potential.

Future studies should focus on the following three aspects to further extend the present findings: (i) For the three preliminarily validated genes, Y1H, dual-luciferase assays, ChIP-seq, and high-throughput Y2H screening will be employed to identify upstream regulators, downstream targets, and interacting partners, thereby constructing a drought-responsive regulatory network and deciphering the molecular basis of the “robust and coordinated” transcriptional response and stomatal “water retention–photosynthesis balance” in ZH1. (ii) For the remaining 12 genes, knockout and overexpression lines will be generated for phenotypic evaluation under varying drought gradients at both seedling and jointing-to-booting stages, combined with cytological and physiological assays, while subcellular localization, transcriptional activity assays, and Co-IP/MS will be conducted to elucidate their molecular functions. (iii) Based on sequence polymorphisms, allelic variation will be screened across *japonica* germplasm, and functional markers tightly linked to drought tolerance will be developed using multi-environment phenotypic data. Through marker-assisted selection, elite alleles from ZH1 will be introgressed into drought-sensitive commercial cultivars, thereby translating fundamental findings into breeding practice.

## 4. Materials and Methods

### 4.1. Plant Materials and Growth Conditions

Seven *japonica* rice (*O*. *sativa ssp. japonica*) cultivars were included in this study, comprising elite hybrids, parental lines, and reference controls; detailed information is provided in Table 1. This design aims to comprehensively analyze the similarities and differences between hybrid rice and its parents in drought response and to highlight the specificity of ZH1 through comparison with other paddy and upland varieties.

The experiment was conducted in rice planting pools at the Rice Research Institute of Yunnan Agricultural University. Each pool measured 130 cm in length and 100 cm in width, with 81 planting holes per pool (9 rows × 9 hills, including border rows). Two water regimes were established: well-watered condition (W) with soil moisture maintained at 70–80%, representing normal irrigation; and water-deficit condition (D) with soil moisture maintained at 40–50%, representing reduced water availability. Each treatment had three experimental replicates.

Seeds were surface-sterilized, soaked, and germinated at 28 °C for 40 h. Direct seeding was performed with five seeds per hole at a depth of 5 cm, followed by covering with a thin layer of soil. Seedlings were thinned to one seedling per hill at 30 days after sowing. Water management followed the requirements for direct-seeded rice nursery cultivation during the first 30 days. After thinning, the water-deficit treatment was initiated by maintaining the field without standing water. Soil moisture content is controlled by using a soil moisture meter. All other field management practices were consistent across treatments.

### 4.2. Phenotypic Characterization

Agronomic trait determination: 90 days after sowing (at the jointing-to-booting stage), was measured using a handheld chlorophyll meter to measure leaf chlorophyll content. After the rice matures, randomly select 5 representative plants from each plot for each variety and treatment to measure plant height (PH), number of tillers (TN), number of leaves (LN), panicle length (PL), panicle neck length (PNL), flag leaf length (FLL), flag leaf width (FLW), effective panicles per plant (EPN), total grains per panicle(TGN), thousand-grain weight (TGW), and seed setting rate (SSR). Seed setting rate (%) = (number of filled grains per panicle/total number of grains per panicle) × 100%.

Stomatal morphology observation: 90 days after sowing, between 9:00 a.m. and 11:00 a.m., fresh, fully unfolded middle segments of sword leaves (about 1 cm × 0.5 cm) from each treated plant were collected. The sample was first gently wiped on the leaf surface with a 70% alcohol cotton ball to remove dust, and then quickly immersed in the FAA fixative (formaldehyde: glacial acetic acid: 70% ethanol = 5:5:90, *v*/*v*/*v*) for fixation for more than 24 h. After the fixed samples underwent ethanol gradient dehydration, critical point drying, and gold spraying by ion sputtering instrument, the stomata morphology of the lower epidermis of the leaves was observed using a scanning electron microscope (SEM, JSM-6390LV, JEOL). Five regions were randomly selected at a 500x field of view using ImageJ software (Version 1.54f). The number of stomata (STN), stomata length (STL), stomatal aperture (STA, the widest distance between stomatal guard cells), and the number of papillae (PAN) in each region were statistically analyzed, and the average values were calculated as the indicators of the sample.

### 4.3. RNA Extraction, Library Preparation, and Sequencing

At 90 days after sowing (simultaneously with stomatal sampling), whole-plant tissues were collected from each treated plant. Five plants per biological replicate were pooled into one sample. Samples were immediately frozen in liquid nitrogen for 3–4 h and then transferred to −80 °C for storage.

Total RNA was extracted using TRIzol Reagent (Invitrogen, Thermo Fisher Scientific, Waltham, MA, USA). RNA concentration and purity (A260/A280 and A260/A230 ratios) were assessed using a NanoDrop 2000 spectrophotometer, and RNA integrity was evaluated using an Agilent 2100 Bioanalyzer. Only samples with an RNA integrity number (RIN) > 7.0 were used for library construction.

Strand-specific mRNA libraries were prepared using the BGI RNA Library Construction Kit. Briefly, mRNA was enriched using Oligo(dT)-conjugated magnetic beads and fragmented at high temperature. The fragments were used as templates for first-strand cDNA synthesis with random primers. Second-strand cDNA was synthesized using dUTP instead of dTTP. After end repair, A-tailing, adapter ligation, and degradation of the U-containing second strand with the UNG enzyme, PCR amplification was performed to obtain strand-specific cDNA libraries. Library quality was confirmed, and sequencing was performed on the BGISEQ-500 platform with a paired-end read length of 150 bp.

### 4.4. Transcriptome Data Analysis and Identification of DEGs

Raw sequencing reads were quality controlled using SOAPnuke (BGI in-house software). Reads containing adapters, with an N ratio > 5%, or of low quality (more than 50% of bases with Phred score < 15) were removed to obtain clean, high-quality reads. Clean reads were aligned to the *japonica* rice reference genome (*Oryza sativa Japonica* Group, IRGSP-1.0) using HISAT2 (v2.1.0) with default parameters. The overall mapping rate was required to be ≥ 85%, and the unique mapping rate ≥ 70%.

Gene expression was quantified using StringTie (v2.1.4) to assemble transcripts and calculate FPKM (Fragments Per Kilobase of transcript per Million mapped reads) values and raw read counts. Differentially expressed genes (DEGs) were identified using the R package DESeq2 (v1.30), which employs a negative binomial distribution model. The significance threshold was |log_2_(fold change)| ≥ 1.0 and false discovery rate (FDR) ≤ 0.05, with FDR adjusted using the Benjamini–Hochberg method.

### 4.5. Functional Enrichment Analysis

Gene Ontology (GO) and Kyoto Encyclopedia of Genes and Genomes (KEGG) pathway enrichment analyses were conducted using: clusterProfiler (v4.0), qvalue < 0.05 as the significance threshold. Enrichment analyses were conducted to identify overrepresented biological processes, molecular functions, and metabolic pathways.

### 4.6. qRT-PCR Validation

To verify the accuracy of the RNA-seq data, 15 candidate genes were ultimately selected for quantitative Real-Time PCR (qRT-PCR) analysis. The method used to extract total RNA was the same as in Section 2.3. Using the TUREscript 1st Strand cDNA Synthesis Kit (Aidlab), 1500 ng of total RNA was reverse transcribed to synthesise the first strand of cDNA.

The qRT-PCR primers (Table S1) were designed using the Primer Premier software (Version 5.0) programme. Use the rice ACTIN gene as the internal reference gene. The PCR reaction was performed using the SYBR Green I dye method on the FQD-96C real-time PCR system (Bio-Rad). The 10 μL reaction system comprised 5 μL of 2× SYBR^®^ Green Supermix, 0.5 μL of each forward and reverse primer (final concentration 200 nM), 1 μL of cDNA template, and 3 μL of ddH_2_O. The reaction procedure was as follows: pre-denaturation at 95 °C for 3 min, followed by denaturation at 95 °C for 10 s and annealing at 60 °C for 30 s, with a total of 39 cycles. Three technical replicates were set up for each sample. Relative gene expression levels were calculated using the 2^−^ΔΔCt method.

### 4.7. Transcription Factor Prediction

Potential upstream transcription factors regulating the identified core water-deficit-responsive genes were predicted using the PlantRegMap database. The analysis identified transcription factors that may bind to cis-regulatory elements within the promoter regions of target genes.

### 4.8. Generation of CRISPR/Cas9 Knockout Mutants

CRISPR/Cas9 knockout vectors targeting *LOC4333842*, *LOC4347618* and *LOC4328441* were constructed. Gene-specific single guide RNA (sgRNA) target sites were designed, and the constructs were transformed into calli of wild-type japonica rice via Agrobacterium tumefaciens-mediated transformation. T1-generation homozygous knockout lines free of Cas9 cassette were screened by DNA sequencing for subsequent phenotypic assays(performed by Wuhan Boyuan Biotechnology Co., Ltd., Wuhan, China Table S2).

### 4.9. Phenotypic Evaluation Under PEG-6000-Simulated Drought Stress

Wild-type and three homozygous knockout seedlings were grown to the three-leaf-one-heart stage. Seedlings were treated with 20% PEG-6000 solution to simulate drought stress. After 48 h of PEG exposure, phenotypic traits including leaf wilting and rolling were evaluated. Multiple biological replicates were set for each genotype.

## 5. Conclusions

In this study, we established a multi-cultivar comparative framework integrating phenotypic and transcriptomic analyses across seven *japonica* rice cultivars under well-watered and water-deficient conditions to elucidate the molecular basis of drought adaptation in the elite hybrid ZH1. Phenotypic analyses revealed that ZH1 maintained relatively stable flag leaf morphology and seed-setting rate under water deficit, indicating superior adaptive capacity. Transcriptomic analysis identified 174 conserved core water-deficit-responsive genes shared across four cultivars, representing a fundamental drought-tolerance regulatory framework. Importantly, through a dual “conserved + specific” screening strategy, we identified 15 core regulators of water-deficit adaptation in ZH1, including 13 cultivar-specific genes and two *japonica*-conserved genes. Phenotypic evaluation of CRISPR/Cas9 knockout lines under 20% (*w*/*v*) PEG-6000-simulated drought stress demonstrated that disruption of *LOC4333842*, *LOC4347618*, and *LOC4328441* significantly increased drought susceptibility, confirming their positive regulatory functions in rice drought responses. These results provide valuable gene resources for molecular breeding aimed at improving drought tolerance in *japonica* rice.

## Figures and Tables

**Table 1:** List of plant materials used in this study.

No.	Cultivar/Line	Code	Remarks	Category
1	Dianheyou 615	ZH1	Elite D1-type three-line *japonica*, hybrid rice F_1_	Paddy-upland dual-adaptation
2	Dianheyou 34	ZH2	Elite D1-type three-line *japonica*, hybrid rice F_1_	Paddy-upland dual-adaptation
3	Nan 34	ZH3	Male parent of ZH2	The restorer line
4	H479B	ZH4	Female parent of ZH1 and ZH2	The maintainer line of the CMS line H479A
5	Nan 615	ZH5	Male parent of ZH1	The restorer line
6	Zhutangxianghangu	ZH6	Local glutinous upland rice	Upland rice control
7	Yungeng 37	ZH7	Elite conventional rice	Paddy rice control

**Table 2:** Agronomic and physiological traits of seven rice cultivars under well-watered and water-deficit conditions.

Var.	Trt.	PH (cm)	TN (No.)	LN (No.)	FLCC (SPAD)	PL (cm)	PNL (cm)	FLL (cm)	FLW (cm)	EPN (No.)	TGN (No.)	SSR (%)	TGW (g)
ZH1	W	120.53±2.54 b	8.00 ± 1.00 ab	9.67 ± 0.58 b	40.73 ± 2.71 c	22.92 ± 2.33 ab	7.29 ± 1.49 ab	34.69 ± 10.01 ab	0.97 ± 0.16 bc	4.00 ± 0.50 ab	165.22 ± 43.71 a	96.61 ± 2.30 a	24.73 ± 0.78 a
ZH2	W	102.93 ± 1.22 c	4.67 ± 2.52 bc	10.00 ± 0.00 b	45.40 ± 1.40 ab	22.07 ± 2.47 ab	8.31 ± 1.10 a	35.84 ± 10.20 ab	0.97 ± 0.16 bc	2.50 ± 1.00 bc	123.33 ± 23.71 bc	95.83 ± 4.10 a	25.47 ± 0.51 a
ZH3	W	95.80 ± 2.52 cd	7.67 ± 3.06 ab	11.00 ± 0.00 ab	44.44 ± 1.60 ab	20.87 ± 2.41 bc	4.60 ± 1.71 cd	27.58 ± 7.00 bc	0.70 ± 0.19 d	3.83 ± 1.53 ab	110.78 ± 17.94 cd	91.63 ± 8.16 ab	23.53 ± 1.00 a
ZH4	W	97.67 ± 3.42 cd	4.00 ± 1.73 c	10.67 ± 0.58 b	46.06 ± 2.44 a	21.13 ± 2.20 bc	7.10 ± 2.07 ab	38.94 ± 7.34 a	1.04 ± 0.05 b	2.00 ± 0.87 c	102.56 ± 25.47 cd	96.73 ± 2.47 a	26.60 ± 1.55 a
ZH5	W	102.90 ± 0.87 c	8.67 ± 2.52 a	12.67 ± 0.58 a	35.50 ± 4.67 d	21.10 ± 1.92 bc	4.20 ± 2.12 cd	30.96 ± 3.93 bc	1.06 ± 0.12 b	4.50 ± 1.00 a	150.56 ± 39.77 ab	87.03 ± 4.38 b	18.52 ± 13.74 b
ZH6	W	142.63 ± 11.36 a	4.67 ± 1.15 bc	11.00 ± 1.00 ab	40.03 ± 3.27 c	25.74 ± 4.44 a	10.18 ± 5.71 a	43.36 ± 15.16 a	1.34 ± 0.13 a	2.33 ± 0.58 bc	146.22 ± 38.44 ab	85.44 ± 12.47 b	27.53 ± 3.20 a
ZH7	W	89.07 ± 1.33 d	5.67 ± 0.58 abc	10.00 ± 0.00 b	42.46 ± 3.86 bc	18.34 ± 1.42 c	6.07 ± 3.41 bc	23.04 ± 8.64 c	0.76 ± 0.14 cd	2.83 ± 0.29 bc	92.00 ± 12.00 d	91.89 ± 4.62 ab	26.57 ± 0.40 a
P(W)	-	0.000 **	0.059	0.000 **	0.000 **	0.000 **	0.004 **	0.001 **	0.000 **	0.029 *	0.000 **	0.002 **	0.491
ZH1	D	92.93 ± 7.37 b	4.67 ± 1.15 ab	12.00 ± 0.00 a	42.59 ± 2.47 bc	21.07 ± 1.77 a	4.04 ± 1.46 a	30.34 ± 2.69 ab	1.15 ± 0.05 b	2.33 ± 0.58 ab	131.78 ± 39.06 a	94.96 ± 4.60 a	22.33 ± 1.89 a
ZH2	D	89.70 ± 5.86 bc	6.00 ± 1.73 a	11.00 ± 0.00 ab	47.44 ± 3.87 a	21.03 ± 2.24 a	4.47 ± 1.53 a	29.84 ± 5.52 ab	1.12 ± 0.09 b	3.00 ± 0.87 a	106.56 ± 19.00 bc	89.63 ± 5.84 ab	20.83 ± 2.53 a
ZH3	D	75.30 ± 1.87 d	5.00 ± 1.00 ab	12.33 ± 1.15 a	44.73 ± 1.13 ab	18.70 ± 1.60 bc	2.07 ± 1.63 bc	25.79 ± 4.70 bc	0.84 ± 0.18 d	2.50 ± 0.50 ab	93.56 ± 19.80 cd	85.43 ± 11.13 bc	19.50 ± 0.87 a
ZH4	D	72.80 ± 5.41 d	3.00 ± 1.73 b	11.00 ± 0.00 ab	43.34 ± 4.61 bc	17.97 ± 1.95 c	3.22 ± 1.81 ab	23.70 ± 2.76 c	1.03 ± 0.12 bc	1.50 ± 0.87 b	69.89 ± 21.22 d	89.06 ± 8.50 ab	21.90 ± 1.37 a
ZH5	D	84.37 ± 5.01 c	4.00 ± 1.00 ab	11.33 ± 0.58 ab	43.16 ± 0.92 bc	18.66 ± 3.44 bc	1.89 ± 1.94 c	26.49 ± 8.00 bc	1.07 ± 0.16 bc	2.00 ± 0.50 ab	118.67 ± 44.32 ab	73.50 ± 14.30 c	23.17 ± 0.91 a
ZH6	D	130.87 ± 6.33 a	6.67 ± 1.53 a	10.67 ± 0.58 b	47.12 ± 4.70 a	24.08 ± 1.51 a	3.06 ± 1.62 abc	29.04 ± 3.86 ab	1.30 ± 0.17 a	3.33 ± 0.76 a	124.89 ± 16.14 ab	78.36 ± 9.47 c	24.50 ± 3.46 a
ZH7	D	75.40 ± 0.36 d	2.67 ± 0.58 b	10.00 ± 0.00 b	45.00 ± 1.30 ab	18.44 ± 2.90 bc	4.01 ± 2.20 a	22.64 ± 5.71 c	0.94 ± 0.18 cd	1.33 ± 0.29 b	96.00 ± 23.00 cd	90.14 ± 9.00 ab	14.79 ± 11.10 b
P(D)	-	0.000 **	0.018 *	0.002 **	0.027 *	0.000 **	0.001 **	0.011 *	0.000 **	0.018 *	0.000 **	0.000 **	0.275

Values are presented as mean ± SD (*n* = 5). Different lowercase letters within the same column and treatment group (W or D) indicate significant differences among cultivars at *p* < 0.05 (Duncan’s multiple range test), values sharing at least one common letter are not significantly different. **, * indicate significant differences at *p* < 0.01 and *p* < 0.05, respectively, as determined by one-way ANOVA for each treatment group. Abbreviations: Var., Cultivar; Trt., Treatment; PH, Plant height (cm); TN, Tiller number (No./plant); LN, Leaf number (No./plant); FLCC, Flag leaf chlorophyll content (SPAD value); PL, Panicle length; PNL, Panicle neck length (cm); FLL, Flag leaf length (cm); FLW, Flag leaf width (cm); EPN, Effective panicle number (No./plant); TGN, Total grains per panicle; SSR, Seed setting rate (%); TGW, Thousand-grain weight (g).

**Table 3:** Stomatal characteristics of seven rice cultivars under well-watered (W) and water-deficit (D) conditions.

Var.	Trt.	STN (Per 500× Field)	STL (3000×, cm)	STA (3000×, cm)	PAN (Per 500× Field)	PAS (Per 3000× Field)
ZH1	W	29.00 ± 2.00 cd	3.57 ± 0.49 a	0.40 ± 0.11 ab	24.67 ± 4.51 b	8.33 ± 0.58 a
ZH2	W	30.67 ± 4.16 bc	3.57 ± 0.06 a	0.47 ± 0.10 ab	31.67 ± 4.04 ab	10.67 ± 0.58 a
ZH3	W	32.33 ± 2.52 b	3.56 ± 0.41 a	0.55 ± 0.09 a	32.33 ± 1.53 ab	7.67 ± 1.15 ab
ZH4	W	39.33 ± 2.08 a	3.11 ± 0.40 a	0.44 ± 0.05 ab	36.00 ± 7.55 a	7.00 ± 1.73 ab
ZH5	W	25.33 ± 0.58 de	3.64 ± 0.37 a	0.38 ± 0.15 b	35.67 ± 2.08 a	5.67 ± 0.58 b
ZH6	W	23.00 ± 5.29 e	3.62 ± 0.30 a	0.52 ± 0.03 a	29.00 ± 6.24 ab	6.33 ± 0.58 b
ZH7	W	36.00 ± 3.61 ab	3.44 ± 0.23 a	0.46 ± 0.10 ab	34.33 ± 9.29 a	7.33 ± 1.53 ab
P(W)	-	0.000 **	0.563	0.49	0.243	0.001 **
ZH1	D	23.00 ± 5.57 a	3.53 ± 0.25 ab	0.44 ± 0.11 a	25.00 ± 2.65 c	7.00 ± 1.73 ab
ZH2	D	27.00 ± 3.46 a	3.27 ± 0.33 bc	0.41 ± 0.05 a	32.67 ± 2.08 bc	7.00 ± 1.00 ab
ZH3	D	29.33 ± 1.53 a	3.46 ± 0.74 ab	0.48 ± 0.06 a	30.33 ± 1.53 bc	8.00 ± 1.00 a
ZH4	D	30.33 ± 4.93 a	3.36 ± 0.35 bc	0.35 ± 0.13 a	34.67 ± 4.62 ab	8.67 ± 0.58 a
ZH5	D	25.33 ± 0.58 a	3.87 ± 0.55 a	0.40 ± 0.10 a	36.00 ± 2.00 ab	6.33 ± 1.15 bc
ZH6	D	27.67 ± 2.31 a	3.99 ± 0.07 a	0.40 ± 0.10 a	27.00 ± 1.73 c	4.33 ± 0.58 c
ZH7	D	25.00 ± 4.00 a	2.43 ± 0.55 d	0.40 ± 0.10 a	41.67 ± 6.11 a	5.67 ± 2.08 bc
P(D)	-	0.244	0.020 *	0.812	0.001 **	0.017 *

Values are presented as mean ± SD (*n* = 5). Different lowercase letters within the same column and treatment group (W or D) indicate significant differences among cultivars at *p* < 0.05 (Duncan’s multiple range test), values sharing at least one common letter are not significantly different. *, ** indicate significant differences at *p* < 0.05 and *p* < 0.01, respectively, as determined by one-way ANOVA for each treatment group. Abbreviations: Var., Cultivar; Trt., Treatment; STN, Stomatal number (per 500× field); STL, Stomatal length (at 3000×, cm); STA, Stomatal aperture (at 3000×, cm); PAN, Papilla number (per 500× field); PAS, Papillae around a stoma (per 3000× field).

## Data Availability

The raw RNA-sequencing data in this study will be deposited in the NCBI Sequence Read Archive (SRA) upon acceptance of the manuscript. All other datasets supporting the conclusions of this work are available within the article and its Supplementary Materials.

## Supplementary Materials

The following supporting information can be downloaded at: https://www.mdpi.com/article/10.3390/ijms27167469/s1.
