# Supplementary material for: Comparative Transcriptomic Analysis of Water-Deficit Responses in Japonica Hybrid Rice ‘Dianheyou 615’
Source: Int J Mol Sci. 2026 Aug 20;27(16):7469. doi: 10.3390/ijms27167469 (PMC13513408; doi:10.3390/ijms27167469)
Supplement: Supplementary file 1 [file ijms-27-07469-s001.zip › Supplementary Figure S1.pdf]

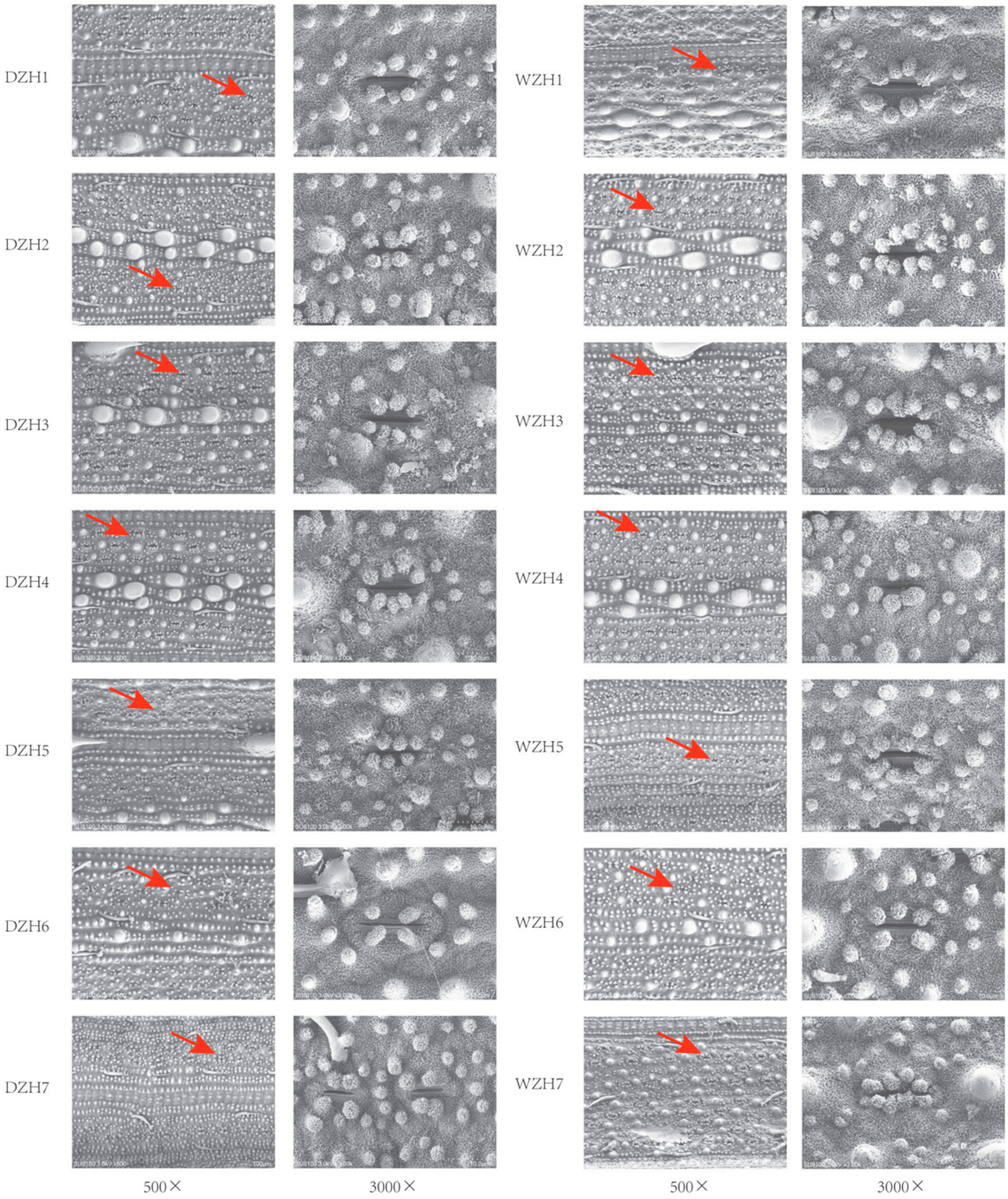

Figure S1. Representative scanning electron microscopy (SEM) images of the flag leaf abaxial epidermis.

Images show stomatal morphology at 500× and 3000× magnifications for seven cultivars under well-watered (WZH1–WZH7) and water-deficit (DZH1–DZH7) conditions. Scale bars are provided in each image. The red arrows indicate silica papillae on rice leaf epidermal surface.
