# Supplementary material for: Comparative Transcriptomic Analysis of Water-Deficit Responses in Japonica Hybrid Rice ‘Dianheyou 615’
Source: Int J Mol Sci. 2026 Aug 20;27(16):7469. doi: 10.3390/ijms27167469 (PMC13513408; doi:10.3390/ijms27167469)
Supplement: Supplementary file 1 [file ijms-27-07469-s001.zip › Supplementary Figure S2.pdf]

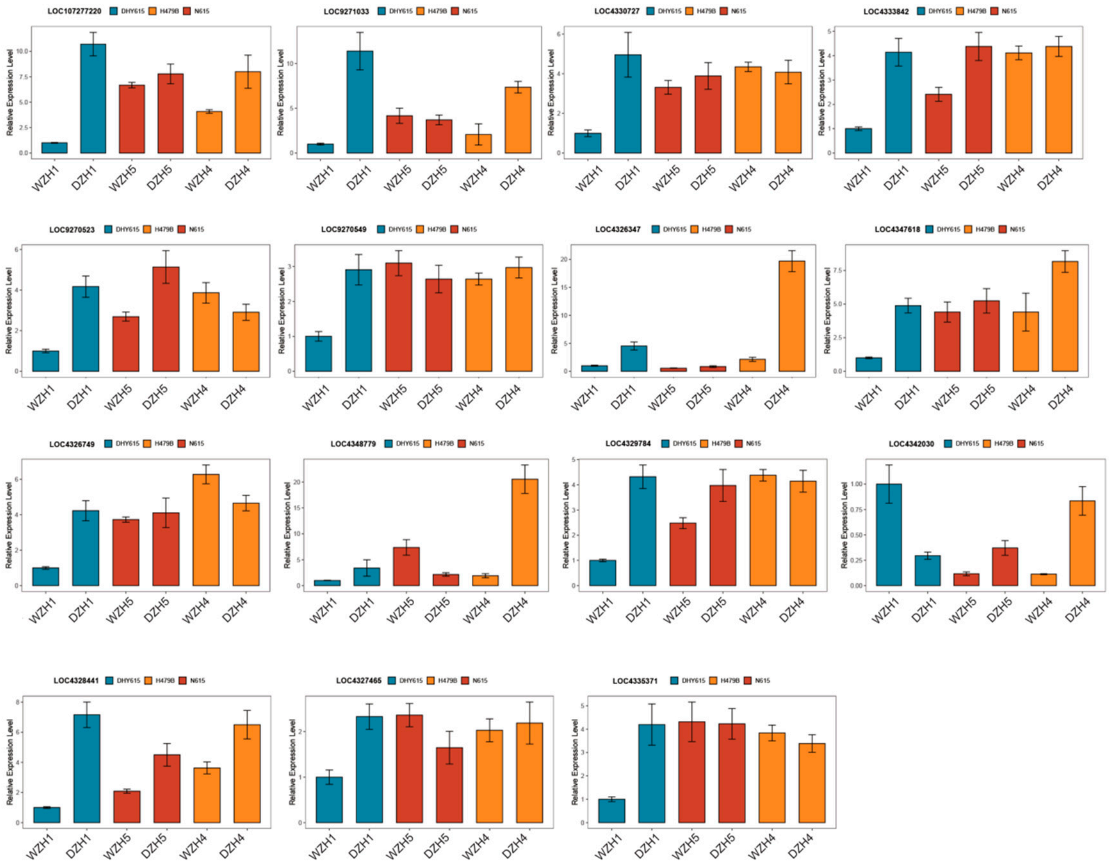

Figure S2. qRT-PCR validation of expression patterns of 15 candidate genes associated with drought response.

Relative expression levels of 15 selected candidate genes were determined by qRT-PCR in 'DHY615' and its parental lines under well-watered (W) and water-deficit (D) conditions.
