# Supplementary material for: Comparative Transcriptomic Analysis of Water-Deficit Responses in Japonica Hybrid Rice ‘Dianheyou 615’
Source: Int J Mol Sci. 2026 Aug 20;27(16):7469. doi: 10.3390/ijms27167469 (PMC13513408; doi:10.3390/ijms27167469)
Supplement: Supplementary file 1 [file ijms-27-07469-s001.zip › Supplementary Table S1.pdf]

Table S1. Primer sequences for qRT-PCR validation.

Gene ID	Primer name	Sequence (5‘→3’)	Tm (℃)
ACTIN	ACTIN-F	TTGCTGACAGGATGAGCAAG	60
ACTIN	ACTIN-R	TGGAATGTGCTGAGAGATGC	60
LOC4335371	1.LOC4335371-F	GCGTCTTCGGTGGATTCT	63
LOC4335371	1.LOC4335371-R	AAGAGGCGGTAGGAGGAG	63
LOC9270549	2-LOC9270549-F	GGCAAGCACCACTTCTTC	63
LOC9270549	2-LOC9270549-R	GGAGACCGCCTTCTTGTA	63
LOC4342030	3-LOC4342030-F	ATCCTATGGCTCTAACCTCTT	63
LOC4342030	3-LOC4342030-R	ACCCTCTTGCTCTTCTCA	63
LOC4326347	4-LOC4326347-F	TGCCGATGTCAAGAGATG	60
LOC4326347	4-LOC4326347-R	CAACCGCTTCCTTCACTA	60
LOC4327465	5.LOC4327465-F	CGGCAAGTTCCATTTTTACG	63
LOC4327465	5.LOC4327465-R	GCTCTTCTCCATCACATCC	63
LOC4330727	6.LOC4330727-F	TGACCTACCACCTCCTTGA	63
LOC4330727	6.LOC4330727-R	TGCTGCCTTGACAATCCA	63
LOC4328441	7.LOC4328441-F	AGTTGGCAGCAGTGTATC	63
LOC4328441	7.LOC4328441-R	TCTCGTATGGACTTGTATTAACC	63
LOC4333842	8.LOC4333842-F	CGGCATTGTCTATGTTGA	63
LOC4333842	8.LOC4333842-R	CTACTTGAGATGGCTGAAC	63
LOC4348779	9.LOC4348779-F	CGAATGCTGAAGGAAATG	63
LOC4348779	9.LOC4348779-R	CCCATATCCACCGTATTG	63
LOC9270523	10.LOC9270523-F	GGCTTCGTTGTTCTCTTG	63
LOC9270523	10.LOC9270523-R	GCACCACCATTGTCATAC	63
LOC4329784	11.LOC4329784-F	ACCTTGTGATTAGCATAGCC	63
LOC4329784	11.LOC4329784-R	CACAGGAACGACGACTTC	63
LOC9271033	12.LOC9271033-F	ACTTCCATACCTACACCATCAT	63
LOC9271033	12.LOC9271033-R	AGTCATCGGCATTCCAGAT	63
LOC4326749	13.LOC4326749-F	CTTGTGCTCCTCTACTTCT	63
LOC4326749	13.LOC4326749-R	TTGAGATTCGCCGTAAGA	63
LOC107277220	14.LOC107277220-F	GAGGGACAGGAAGGTGGC	63
LOC107277220	14.LOC107277220-R	ATCTCGGCGACGACTCTG	63
LOC4347618	15.LOC4347618-F	AACGATGGCGACGAAGAAGA	63
LOC4347618	15.LOC4347618-R	ATCCCGCCGAACCTGAAC	63
