# Supplementary material for: Comparative Transcriptomic Analysis of Water-Deficit Responses in Japonica Hybrid Rice ‘Dianheyou 615’
Source: Int J Mol Sci. 2026 Aug 20;27(16):7469. doi: 10.3390/ijms27167469 (PMC13513408; doi:10.3390/ijms27167469)
Supplement: Supplementary file 1 [file ijms-27-07469-s001.zip › Supplementary Table S2.pdf]

Table S2. Target sequences and sequencing-based mutation identification of CRISPR/Cas9 knockout lines for three candidate japonica rice genes.

<table>
<thead>
<tr><th>Line ID</th><th>Gene LOC ID</th><th>MSU locus ID</th><th>Target Sequence</th><th>Chromosome Mutation Count</th><th>Mutation Result</th><th>Read Count</th><th>Edited sequence</th><th>Editing Type</th></tr>
</thead>
<tbody>
<tr><td>L187 [M132681]-5</td><td>LOC4333842</td><td>Os03g0703100</td><td>GCCTGAATTTCGATGCATATCGG;CATATCGGTTTTCAATCTCGTGG</td><td>2</td><td>CGATGCATATCGGTTTTCAA--TCGTGGTCAAGGATT(|delete|) || CGATGCATATCGGTTTTCAA--TCGTGGTCAAGGATT(|delete|)</td><td>8428</td><td>ATGAATTTCGGCAGCTTGATAATTGCTTACTTTTTTTTTTTCTGAAAGAAGATAATTACTTACATTGTCTTCTTGCTAGCACAGGAAGATGTTGATCTCTTGAAAAGCCTGAATTTCGATGCATATCGGTTTTCAATCGTGGTCAAGGATTTTCCCTGGTAAATAAAAATATGGCTCTGGCTATAGCATCTACTTGTACTGGCTAGCTGGATGAGGATG</td><td>Homozygous Knockout</td></tr>
<tr><td>L176 [M132328]-34</td><td>LOC4347618</td><td>Os09g0522000</td><td>CGAGGCGTTCCAGCGCCAGTCGG</td><td>2</td><td>GCCGTCGAGGCGTTCCAGCG-CAGTCGGCCGCGCCG(|delete|) || GCCGTCGAGGCGTTCCAGCG-CAGTCGGCCGCGCCG(|delete|)</td><td>16055</td><td>CCTCAACTTCGCCGACTCCGCGTGGCGGATGCCGCCCGTCCCCGCGTCCGCCGCGCTCGCCGGCGCGAGGGGGGTCAGGGACGCCGTCGCCGTGGCCGTCGAGGCGTTCCAGCGCAGTCGGCCGCGCCGTCGTCTCCGGCGGAGACCTTCGCCAACGATGGCGACGAAGAAGAAGACAACAAGGACGTGTTGCC</td><td>Homozygous Knockout</td></tr>
<tr><td>L177 [M132323]-8</td><td>LOC4328441</td><td>Os02g0170100</td><td>TGACACTCATGAATGGGTTAAGG;GGCATGGTCTGTAATTCCAACGG</td><td>2</td><td>ATGCTGACACTCATGAATGG------------------------------GGAATTACAGACCAT(|delete|) || ATGCTGACACTCATGAATGG------------------------------GGAATTACAGACCAT(|delete|)</td><td>47804</td><td>CATCTGATGAAGTTGTGCATATTGCTTGTTTTCTATTTTTTTCAGTGCTGAAGGATCTGAAGTATGCTGACACTCATGAATGGGGAATTACAGACCATGCCCAGGTTGATCTATTCATACGTTATATCACATTCATTGCTTCATTCTGTACATACGCAT</td><td>Homozygous Knockout</td></tr>
</tbody>
</table>
